# Review of Current Spinal Robotic Orthoses

**DOI:** 10.3390/healthcare9010070

**Published:** 2021-01-13

**Authors:** Siu Kei David Mak, Dino Accoto

**Affiliations:** 1 Department of Neurosurgery, National Neuroscience Institute, Singapore 308433, Singapore; 2 School of Mechanical & Aerospace Engineering, Nanyang Technological University, Nanyang, Singapore 639798, Singapore; daccoto@ntu.edu.sg

**Keywords:** osteoporotic spine fracture, spinal orthosis, exoskeleton, wearable robotics, active orthosis

## Abstract

Osteoporotic spine fractures (OSF) are common sequelae of osteoporosis. OSF are directly correlated with increasing age and incidence of osteoporosis. OSF are treated conservatively or surgically. Associated acute pain, chronic disabilities, and progressive deformities are well documented. Conservative measures include a combination of initial bed rest, analgesia, early physiotherapy, and a spinal brace (orthosis), with the aim for early rehabilitation to prevent complications of immobile state. Spinal bracing is commonly used for symptomatic management of OSF. While traditional spinal braces aim to maintain the neutral spinal alignment and reduce the axial loading on the fractured vertebrae, they are well known for complications including discomfort with reduced compliance, atrophy of paraspinal muscles, and restriction of chest expansion leading to chest infections. Exoskeletons have been developed to passively assist and actively augment human movements with different types of actuators. Flexible, versatile spinal exoskeletons are designed to better support the spine. As new technologies enable the development of motorized wearable exoskeletons, several types have been introduced into the medical field application. We have provided a thorough review of the current spinal robotic technologies in this paper. The shortcomings in the current spinal exoskeletons were identified. Their limitations on the use for patients with OSF with potential improvement strategies were discussed. With our current knowledge of spinal orthosis for conservatively managed OSF, a semi-rigid backpack style thoracolumbar spinal robotic orthosis will reduce spinal bone stress and improve back muscle support. This will lead to back pain reduction, improved posture, and overall mobility. Early mobilization is an important part of management of patients with OSF as it reduces the chance of developing complications related to their immobile state for patients with OSF, which will be helpful for their recovery.

## 1. Background

At present, 125 million people are aged 80 years or older. By 2050, there will be almost 434 million people in this age group worldwide. Between 2010 and 2060, the number of people aged above 65 years will increase from 17.4% to 29.5% of the total population [1]. One in two women and one in five men over 50 years of age will suffer an osteoporotic fracture. During the year 2000, there were an estimated 9 million new fragility fractures worldwide, of which 1.4 million were symptomatic vertebral fractures [2]. Osteoporotic spine fractures (OSF) are more common in women compared to men (a 4:1 ratio). They cause severe pain, disability, deformities, and even death. This has a significant impact on their activities of daily living (ADLs) and quality of life (QOL) [3,4]. Hence, OSF are gaining well-deserved recognition in the medical field [5]. Osteoporotic fractures exert a tremendous burden on older people and healthcare budgets.

### Biomechanics of the Spine

**Figure 1:**
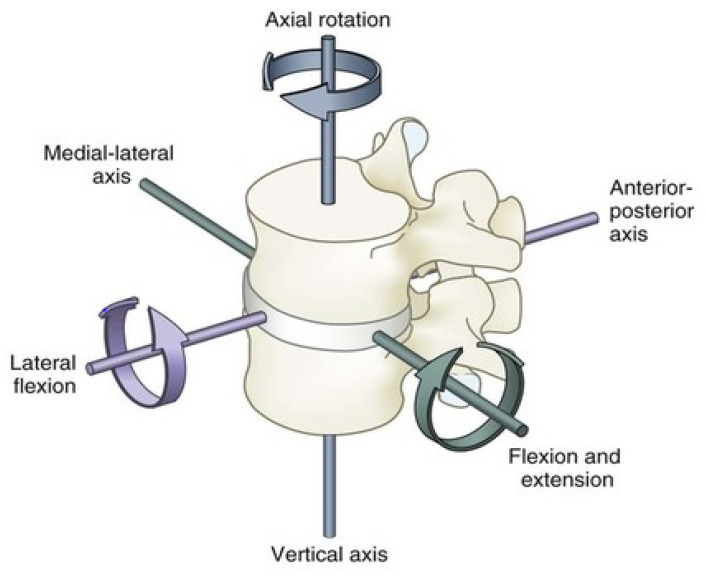
Standard range of movements for the human lumbar spine.

The human spine, with the lumbar region in particular, carries three major motions: sagittal plane flexion/extension, lateral bending, and axial rotation, of which the sagittal plane flexion/extension is the most significant in terms of the range of movement (ROM, see Figure 1).

The overall ROM of the lumbar spine itself is usually supported by two main groups of muscle. We may classify them as the anterior and the posterior groups of muscle. Models of the human spine suggest that the lumbar spine can be modeled as one additional joint in the contribution for flexion and extension on the sagittal plane [6]. Lumbar spine ROM parameters are as follows: flexion 40–60 degrees, extension 20–35 degrees, lateral bending 15–20 degrees, and axial rotation 3–18 degrees.

The posterior spinal muscle group consists of a thick superficial layer of thoracolumbar fascia, which is attached to the spinous processes and the supraspinous ligaments. It extends bilaterally to cover the erector spinae muscles (the iliocostalis, longissimus, spinalis muscles). Beneath this thick fascia lies the erector spinae muscles which arise from the sacrum, iliac crest, and the spinous processes of the thoracolumbar spine. They act as part of the posterior column. Together with the facet joint and posterior ligamentous complexes, these muscles form the posterior tension band for spinal stabilization, and, most importantly, they are also the main muscles for thoracolumbar spine ROM.

**Figure 2:**
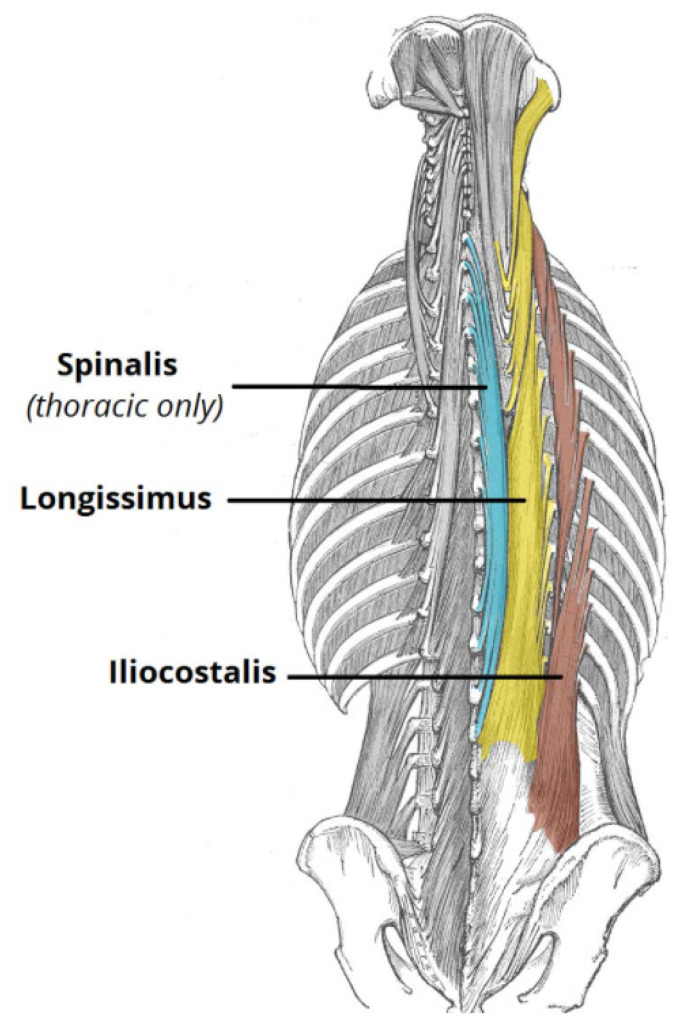
The principle posterior spinal muscles.

The anterior group of spinal muscles consists of the quadratus lumborum and the psoas major muscles. They do not contribute to the overall spinal stability, but they help with ROM of the spine. Hence, the posterior muscle group is considered the principle muscle group for thoracolumbar spinal movements (see Figure 2).

Human vertebral bodies and intervertebral discs together are designed to resist large compression forces (ranging between 2 N and 14,000 N), while the shearing forces are mostly loaded onto the posterior facet joints (ranging between 600 to 2800 N). Each lumbar intervertebral disc can also bear shearing forces between 380 and 760 N [7].

The mean back extensor strength of human lumbar spine decreases with age, and it measures from 284 N, to 267 N, 262 N, and 172 N for females in age groups between 40–49, 50–59, 60–69, and 70–79, respectively [8]. The posterior spinal muscles play a crucial role in the posterior tension band that maintains our normal posture by balancing the biomechanical tendency of the spine to fall forward. This function coincidentally reduces mechanical stress on the vertebral bodies [9].

There are various biomechanical models which have been developed to estimate lumbar spinal loading and forces [10]. Recent studies estimated these forces during symmetric lifting and asymmetric lifting tasks using a whole-body musculoskeletal modelling approach and found that torsion of the spine during asymmetric lifting required more trunk muscle contractions than symmetric lifting. Lifting with a twisting movement of the spine increased the three-dimensional lumbar spinal forces at the L5/S1, L4/L5, and L3/L4 joints from 3000 N to 5000 N. The torque for asymmetric lifting was also greater and up to 180Nm for a 12kg load [11,12].

Flexion-relaxation of the posterior spinal muscles occurs in positions of quiet standing with substantial trunk flexion. One study found that the myoelectric activity (EMG) is diminished on 40 degrees flexion of the lumbar vertebrae, and it is nearly absent in quiet standing at maximum flexion, i.e., no further muscle contractions are required. The forces required to maintain the equilibrium at the full range flexion, provided by the passive muscle tissue, tendons, and ligaments, were measured to be 700 N [13].

## 2. Passive Spine Braces in the Treatment of Osteoporotic Spine Fractures (OSF)

Treatment of OSF can be classified as surgical or non-surgical, depending on the morphology of the fracture pattern as well as the clinical condition of the patient. Stable fracture patterns include those of vertebral body compression fractures which involve only the anterior spinal column while the posterior muscular–ligamentous complex remains intact [14,15]. These stable OSF are treated non-surgically.

**Figure 3:**
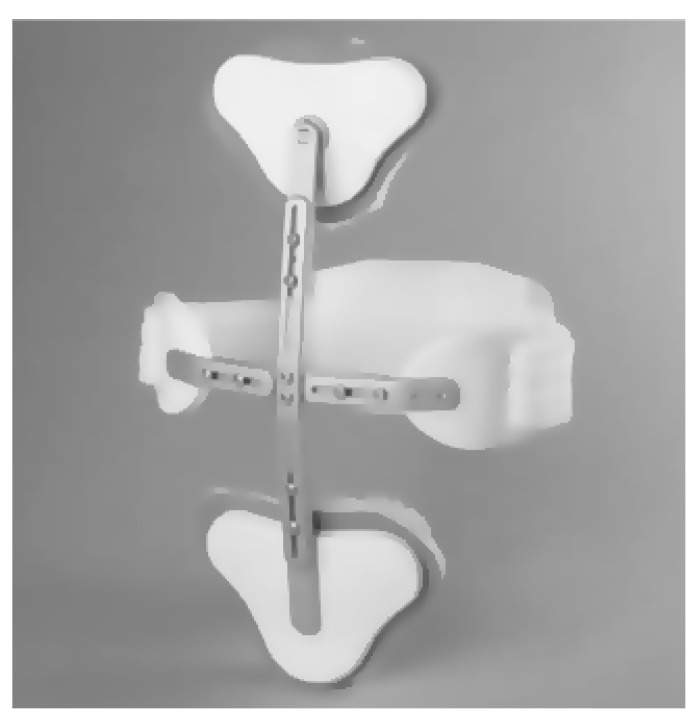
Adapted from Trulife—C.A.S.H. Orthosis.

The start of non-surgical treatment for stable OSF usually consists of initial bed rest, analgesia, early physiotherapy, early rehabilitation, and a rigid spinal brace (orthosis). Pain related to the OSF may last up to 4 months [16]. Spinal braces are usually applied for a minimum of 3 weeks and up to 12 months, depending on the patient’s symptoms and the fracture healing progress [17,18,19,20]. Patients are encouraged to apply the spinal brace at all times unless they are lying down, for example, during bedrest. Typically, these braces are usually fixed and rigid (for example, TLSO, thoracolumbar spinal orthosis, see Figure 3) [21].

Progressive vertebral body collapse is a known complication for patients with OSF. It is a potential complication due to lack of proper spinal support. This may eventually result in progressive kyphotic deformity of the spine. Radiologically, this is regularly seen as worsening kyphosis of the spine on follow-up, when compared to initial radiological images taken at time of injury. Hyper-kyphosis of the spine itself imposes further stress on the anterior spinal column. This increases the risk of new fractures and disrupts normal balance, thereby increasing the risk of falls and, as a result, other injuries and fractures [22]. The most important point to note is that progressive vertebral body collapse with kyphotic deformity may result in conversion of a benign OSF into a burst fracture type pattern, which will lead to neurological compromise from a spinal cord with cauda equina compression.

**Figure 4:**
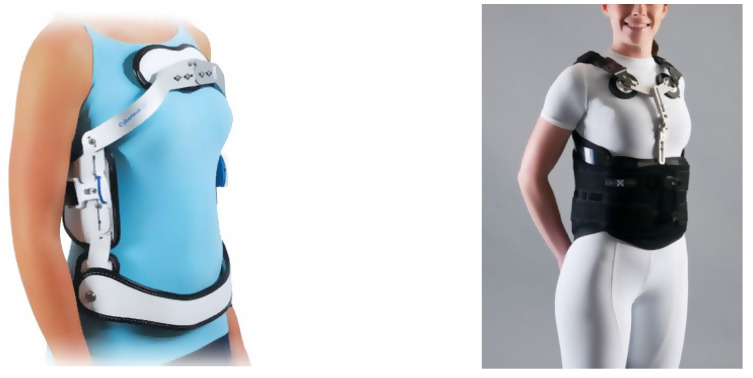
(**Left**): Adapted from Alberta Association of Orthotists and Prosthetists. (**Right**): Adapted from the Steeper Group—Oasis Spinal Brace.

The goals of traditional orthotic treatment involve a combination of limiting motion and correcting posture, thus reducing pain and fatigue, and improving overall function or participation at rehabilitation. A spinal brace in OSF can prevent pain from truncal movements by stabilizing the spine [23] through axial spinal support and reducing the effort of the para-spinal muscles. This in turn will promote fracture healing [24] (see Figure 4). The effects of these braces on the stabilization of the injured vertebral body have been demonstrated by previous biomechanical studies [9,25,26]. In the meantime, early (within a few days of injury, if possible) mobilization of this group of patients is recommended, as it will significantly avoid the complications of prolonged bedrest [27].

As mentioned above, the posterior spinal muscles are an important part of the human posterior tension band, which maintains the truncal posture. Thus, strengthening the posterior spinal muscles will improve lumbar lordosis and posture and will reduce acute fracture pain as well as chronic back pain associated with kyphotic deformity from progressive vertebral body collapse [18,28]. This reinforcement is especially important since axial musculature decreases in strength with age, particularly among women, who are most at risk for OSF [29]. Studies have also demonstrated that back extension strength and lumbar mobility are the most important factors for quality of life compared to other relevant factors such as lumbar kyphosis angle and bone mineral density, especially in females with osteoporosis [30].

### Limitations with Traditional Spine Braces in OSF

While rigid spinal braces are widely prescribed in the conservative, non-surgical management of OSF, there is a lack of high-quality evidence for their effectiveness. Traditional rigid spine braces aim to restore the normal curvature of the spine by applying a set of displacements at different levels of the torso, which are typically achieved by adjusting the geometry of the brace or adding soft pads inside the brace. This process relies mostly on bi-planar radiographs of the spine taken in the sagittal and coronal planes, surface topography of the torso, and the experience of the orthotist who has limited quantitative data.

A recent meta-analysis looking at the effect of a brace on OSF concluded that a Spino-med orthosis, which work with a concept of improving the strength of users’ trunk muscle, unlike traditional rigid braces which offer immobilization of the spine, may subsequently reduce pain, reduce kyphosis angle deterioration, and improve quality of life [16]. However, due to the high risk of bias in the blinding process and outcome assessment in the included studies, only low-quality evidence proved using Spino-med braces could bring large and significant beneficial effect to patients with subacute osteoporotic vertebral fractures [18]. Spinal braces are part of the conservative, non-surgical care regime for conservatively managed OSF and have been compared to external support provided by a variety of orthoses or casting. As a result, it is not possible to properly quantify a comparison between the designs, and it makes the true treatment effect of an orthosis difficult to elucidate. There are also wide variations in terms of outcome measurements and the duration of brace application in different studies. Currently, there is no study which evaluates the long-term outcomes of the traditional rigid spinal brace application.

Although spinal braces for OSF are known to improve trunk muscle strength, posture, and body height, all which will improve quality of life and ability to perform activities of daily living (ADL) for patients with OSF [30], these rigid spinal braces have a number of limitations due to their rigid, static, and sensorless features. The rigidity of the brace makes it difficult to wear over extended periods of time, and it interferes with ADLs. User compliance will subsequently become a main issue. The braces statically hold the torso in a set posture without detection of the forces/moments exerted on the torso. These excessive forces are known to cause skin breakdown, pressure sores, and even worsening of spinal deformities [31,32]. The lack of freedom of control of the correction provided by the brace at the coronal and sagittal planes makes it difficult to adapt to natural changes of the spine and may impair healing and lead to diminished effectiveness. Non-static braces have been designed to address some of these limitations. However, non-static braces are only useful for temporary relief of symptoms. As they do not provide spinal support, their function in preventing progressive kyphosis, for example in patients post-OSF and especially at the junctional thoracolumbar levels, is known to not be effective [16].

A systemic review of spinal braces in OSF showed no evidence to support the regular use of rigid TLSO [33]. Potential downfalls of a rigid spinal brace such as TLSO are well documented in literature. As mentioned above, these include patient discomfort, which may decrease compliance. There are also risks for skin breakdown if the brace edges are not carefully padded. In addition, a brace that is too restrictive may impede the patient’s respiratory volume and effort and may eventually lead to chest infections. With prolonged periods of bracing, there is also a high chance for deconditioning and atrophy of the trunk and erector spinae muscles, which in turn will lead to increased risk of future falls and further injuries and/or fractures [16,21,29,33,34].

A semi-rigid backpack or bell-style thoracolumbar orthosis (TLO) could reduce pain and improve participation in activities of daily life [24]. A weighted orthosis seems to improve balance, while there is little to no current evidence to suggest that bracing will negatively affect wedging and kyphotic progression of the spine in OSF [17,33].

## 3. Active Spine Braces

Exoskeletons provide a viable solution to externally assist human movement. To address the weaknesses of traditional spinal braces, researchers have proposed flexible, versatile spinal exoskeletons with the potential to better support the spine. As manual therapy on traditional rigid braces seems to be physically demanding for both the patient and therapist, the exoskeletons can increase the efficiency of rehabilitation therapy, while providing more intensive patient training, better quantitative feedback, and eventually improved overall functional outcomes for patients compared to traditional therapies alone.

### 3.1. State of the Art

(i) Classification of Devices: Active vs. Passive

Wearable robotic exoskeletons are typically described as active or passive types.

An active exoskeleton comprises one of more actuators that augments the human’s power and helps in actuating the human joints. These actuators may be electric motors, hydraulic actuators, pneumatic muscles, or other types.

A passive exoskeleton system does not use any type of actuator but rather materials, springs, or dampers with the ability to store energy harvested by human motion which is then used as required to support a posture or a motion.

Active types of wearable robotic exoskeletons may potentially unload more joints at a time, but the design usually involves more actuators/motors, which drastically increases the weight of the whole exoskeleton. It is also difficult to fully replicate the true movements of human joints, and this poses a technical challenge for anthropomorphic active exoskeletons to reflect the human anatomy, kinematics, and kinetics and to enable natural, and hence comfortable, movements. For example, our knee joints may also form a challenge as the center of rotation shifts during flexion. Moreover, rotational movement in any joint requires movement between the skin and skeletal structures. To accommodate this ability of tissue stretching during movement, the exoskeleton should ideally be designed so that it is able to extend or shorten, especially during rotational movements.

(ii) Support of Different Body Parts

An exoskeleton can be labelled in terms of the body part that it was designed to support. It may provide power or support to the spine (referred as spinal or truncal exoskeletons), lower limbs (referred as lower body exoskeletons), upper extremities (referred as upper body exoskeletons), and both upper and lower extremities (so called full-body exoskeletons).

(iii) Human Anthropometry

Exoskeleton designs may also be classified in terms of how an exoskeleton fits or resembles the human anthropometry. Anthropomorphic exoskeletons have exoskeleton joints with movement axes that are aligned with the movement of human joints. Hence, a fully anthropomorphic type of exoskeleton enables the robot to make the same motions as the operator, thus offering a large freedom of motion. However, it is challenging to develop an anthropomorphic system which can fit different sizes of users while accommodating their natural body habitat and movements at the same time.

On the other hand, non-anthropomorphic types are generally simpler as compared to their counterparts, and they can be designed to have an optimized structure for specific tasks, allowing more effective energy consumption.

(iv) Early Exoskeletons

**Figure 5:**
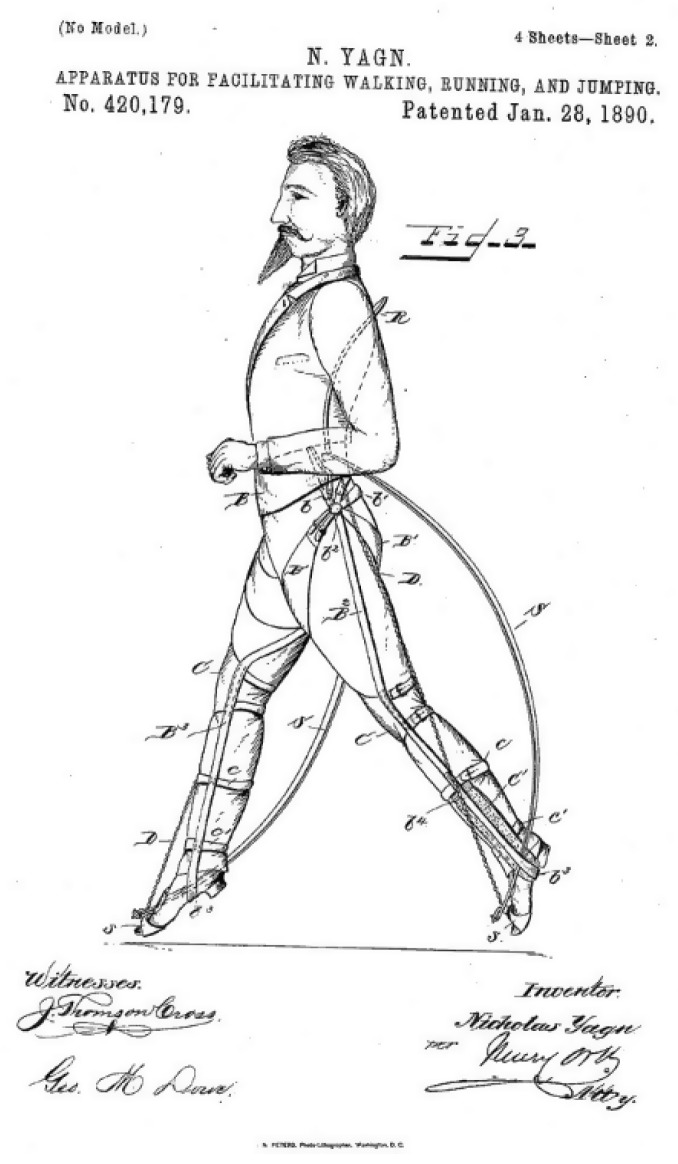
First patented exoskeleton.

Powered exoskeletons did not start as assistive devices. The first patent for an exoskeleton, filed by a Russian inventor named Nicholas Yagn, was approved in January 1890 [35]. This design of an “apparatus for facilitating walking” involved long springs attached to each leg, designed to give soldiers in the Russian Army an advantage when running (see Figure 5).

**Figure 6:**
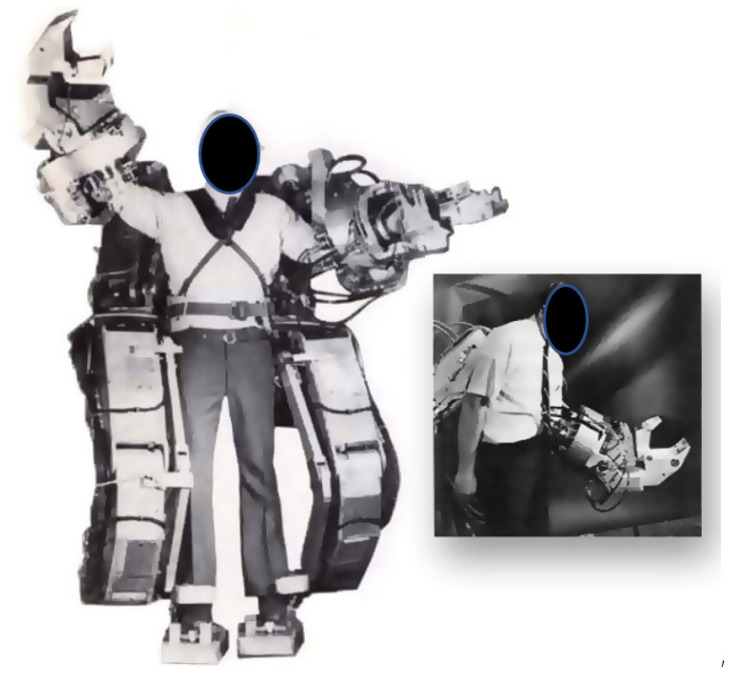
The ‘Pitman’ exoskeleton.

In the 1960s, researchers began to create elaborate powered exoskeletons, as demands were high from the military. The “Hardiman” is an enormous full-body hydraulically-powered exoskeleton prototype weighing 680 kg which was designed to amplify the strength and endurance of human arms and legs [36,37] (see Figure 6).

In the mid-1980s, the “Pitman,” a design concept for augmenting the capability of soldiers, emerged [38]. An independent researcher expanded on the Hardiman and Pitman concepts in a concept paper by incorporating singularity-free, pitch–yaw type joints in order to present a full-body, 26 DOFs exoskeleton concept [39].

**Figure 7:**
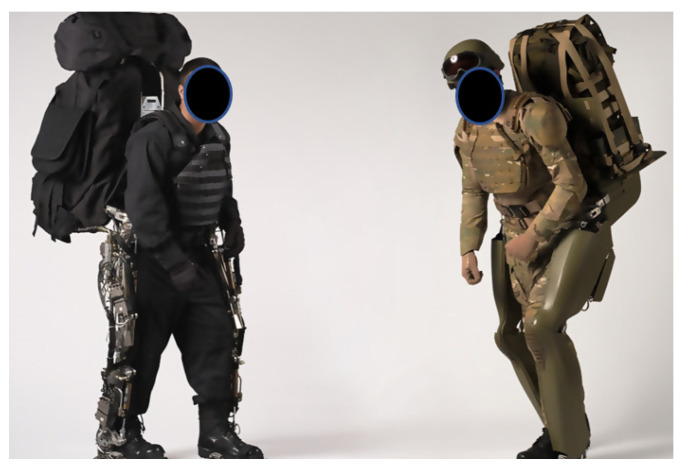
The BLEEX exoskeleton.

It was not until the 2000s that exoskeletons for rehabilitation and military usage became a reality. An early example is the Berkeley Lower Extremity Exoskeleton (BLEEX), an active exoskeleton designed for military load-carrying [40]. The exoskeleton is actuated via bidirectional linear hydraulic cylinders. BLEEX consumes an average of 1143 W of hydraulic power during level-ground walking, as well as 200 W of electrical power for the electronics and control systems [41,42] (see Figure 7). In contrast, a similarly sized, 75 kg human consumes approximately 165 W of metabolic power during level-ground walking [43].

**Figure 8:**
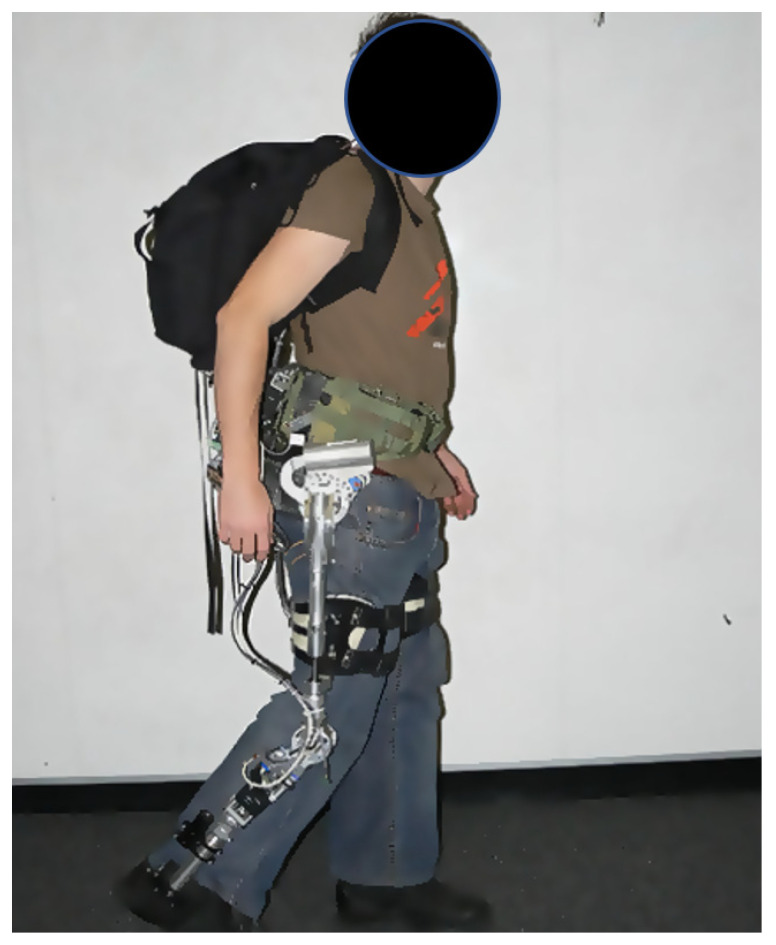
The MIT exoskeleton.

The MIT exoskeleton is an example of a passive exoskeleton which employs a quasi-passive design which relies completely on the controlled release of energy stored in springs during the (negative power) phases of the walking gait. It also consists of a magnetorheological variable damper (motion in the flexion/extension direction) that controls the dissipation of energy at appropriate levels throughout the gait cycle. However, metabolic studies with the quasi-passive exoskeleton showed a 10% increase in walking metabolic expenditure [44,45] (see Figure 8).

(v) Application of Exoskeletons

Various powered exoskeletons are being applied in the medical, military, civilian, and industrial fields. For application in the medical field, these are a few examples where powered exoskeletons are used. This includes in rehabilitation for post stroke and spine injury patients, for example, to improve quality of life and function. The ability of exoskeletons to augment power and durability may help allied health professionals to carry patients or lift heavy objects. Some exoskeletons are developed with the aim for instrumented movement analysis with sensor-based measurement techniques, which will allow objective description and quantitative assessment of motor functions and motor abilities.

A review paper evaluated the potential effects on physical loading for both passive and active industrial exoskeletons. It concluded that the different types of exoskeletons varied greatly in terms of the part of the body being supported (either lower limbs, upper limbs, or full body), the choice of main framework materials, and the actuation types. It also highlighted that supporting literature showed up to 60% average reduction in electromyography (EMG) signals at the posterior spinal muscle group, as well decreasing muscle activity in the lower extremities (e.g., during stairclimbing), shoulders, and upper extremities for different types of hand and arm work [45].

**Figure 9:**
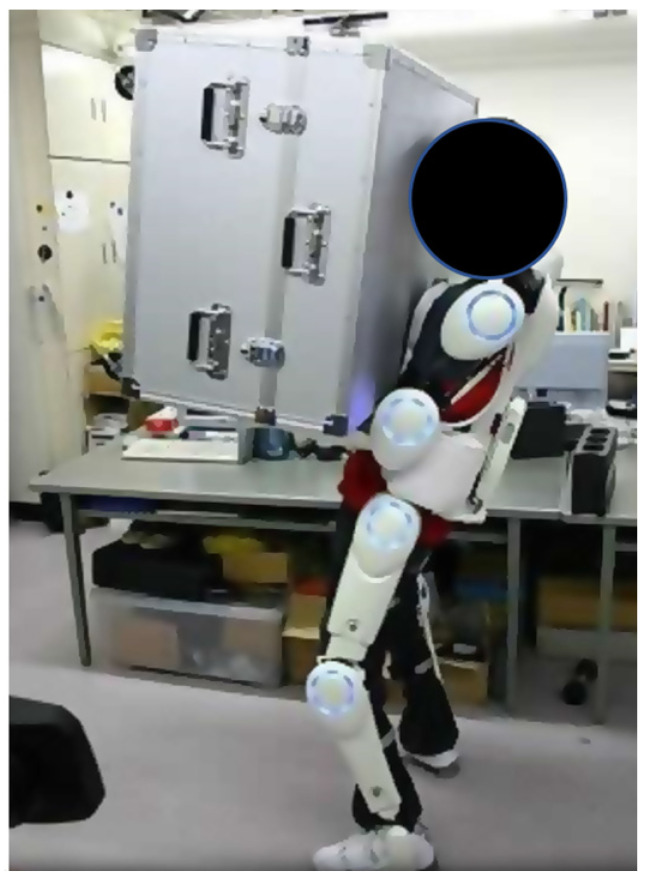
Men wearing the HAL carrying heavy load.

A more recent exoskeleton concept is the Hybrid Assistive Limb (HAL) that was designed both for healthy individuals and impaired individuals to augment their abilities with a wearable suit, capable of locomotive gait assistance via neuro-electromyographic stimuli that drive the exoskeleton use. The HAL powers the flexion/extension joints at the hip and knee via a DC motor. This is different from the earlier designs of BLEEX and MIT exoskeleton, as these exoskeletons work to transfer the load onto the ground surface, while the HAL design, on the other hand, directly augments the torques of the lower limb joints [46] (see Figure 9).

**Figure 10:**
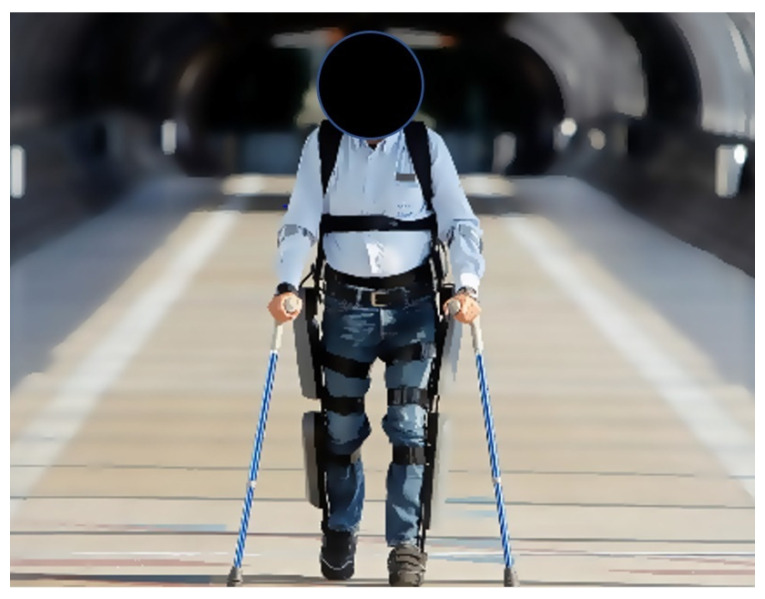
The ReWalk system.

Common clinical uses of exoskeletons are those developed to aid disabled operators, such as paraplegic survivors from spinal cord injuries. One example is the ReWalk, developed by Argo Medical Technologies Ltd. [47]. The ReWalk is FDA approved in the US for home use for individuals with lower limb paraplegia from spinal cord injury. It is a battery-powered hip–knee exoskeleton and requires intact upper limbs as users need to use crutches to maintain their balance. There are sensors in the chest detecting the angle of the torso and the shift in user’s center of gravity. Although the ReWalk system weighs 21 kg, it carries its own weight, and the user only feels the weight of the backpack, which is approximately 2.3 kg (see Figure 10). Mekki et al. have provided a thorough review of the current robotic exoskeletons for the survivors from acute spinal cord injury [48].

### 3.2. Overview of Spinal Exoskeletons

(i) Current Spinal Exoskeletons

There are a few researchers who have proposed and designed various spinal exoskeletons that can perform mechanical adjustments at multiple spine levels and in multiple movement directions [49,50,51,52]. These spinal exoskeletons, however, have all been designed with the aim of providing back support to reduce the incidence of lower back pain, which causes significant morbidity of workers worldwide. These spinal exoskeletons are either passive or actively augmented with motors, potentially providing activity-specific spinal support that can be specifically tailored to complement user-specific activities [53]. These flexible designs mean that spinal exoskeletons themselves have a huge advantage in lower back support when compared to the traditional rigid spinal braces.

Spinal back-support exoskeletons can be divided into two categories in terms of their overall framework design: soft exo-suits and rigid exoskeletons. Rigid exoskeletons can also be classified as active or passive. As discussed above, passive designs are mainly used to reduce the burden on the user rather than amplify the wearer’s forces. While active devices are more powerful, they are also heavier as they require actuators and a power source.

Examples of soft Exo-suits, which will be presented in more details, are the Personal Lift Augmentation Device (PLAD) and the Smart Suit Lite (SSL) which use plastic elements to store energy to support the lumbar spine. On the other hand, devices such as the Bending Non-Demand Return (BNDR) and LAEVO transmit forces through rigid plates or stiff beams, while the Back-Support Muscle Suit uses McKibben artificial muscles as actuators for support during weightlifting tasks.

**Figure 11:**
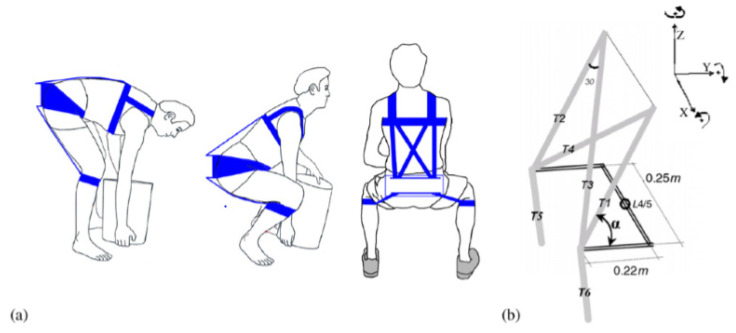
(**a**) the PLAD (**b**) Schematic of the upper body plastic bands with elements under tension (T, 1–6). (Copyright Springer Nature).

The PLAD uses elastic elements that are applied parallel to the posterior spinal muscles. The plastic bands, made of simple Thera-Band^®^ Latex, store energy as they are stretched during bending of the trunk in tasks such as lifting. The saved energy is released on the reverse phase of the action [54]. The PLAD can reduce posterior spinal muscle EMG during lifting tasks by up to 40%, with a 23–29% reduction of lumbar spine compressive forces [55]. Depending on the lifting activity, the reduction of posterior spinal muscle activity is accompanied by an increased effort of some of the lower limb muscles, namely the biceps femoris [56]. This is likely the result of a transfer of load to the lower limbs. Muscle fatigue in the EMG signal, defined as the combination of amplitude increase and frequency content decrease, is significantly less in the case of prolonged repetitive lifting and lowering over 45 min with the application of the PLAD, as compared to 10 min without application of PLAD. The PLAD’s reaction forces with regards to the pelvic girdle contact force ranged from 221.3 to 468.5 N [56] (see Figure 11).

**Figure 12:**
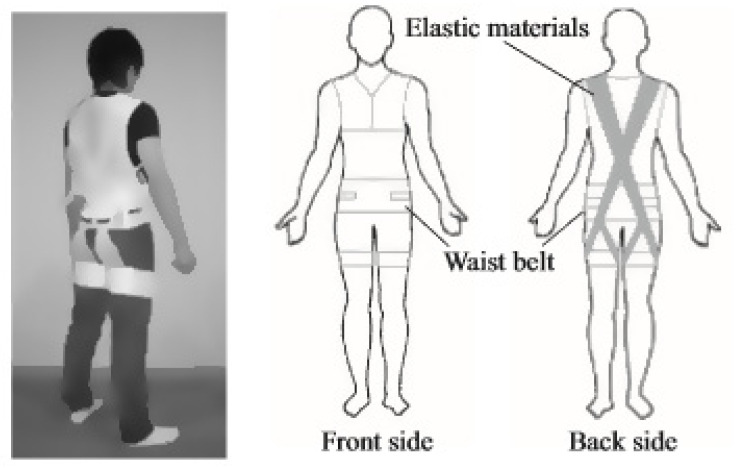
The Smart Suit Lite (SSL).

The Smart Suit Lite (SSL) was developed to reduce lumbar muscular loading during nursing care. This passive assist device uses elastic belts connected with pulleys. It utilizes the elastomeric force of the elastic materials to assist the user. The belts exercise an assistive torque on the lumbar joint and on the hip joint when the wearer changes posture during heavy load lifting. This is augmented by an additional elastic belt around the torso for stabilization. This design works by transferring the force into the pelvis while providing lumbar support and increasing intra-abdominal pressure. In the process, it reduces the lumbar intervertebral disc pressure and the work required from the lumbar posterior spinal muscles by 24.4% [57] (see Figure 12).

**Figure 13:**
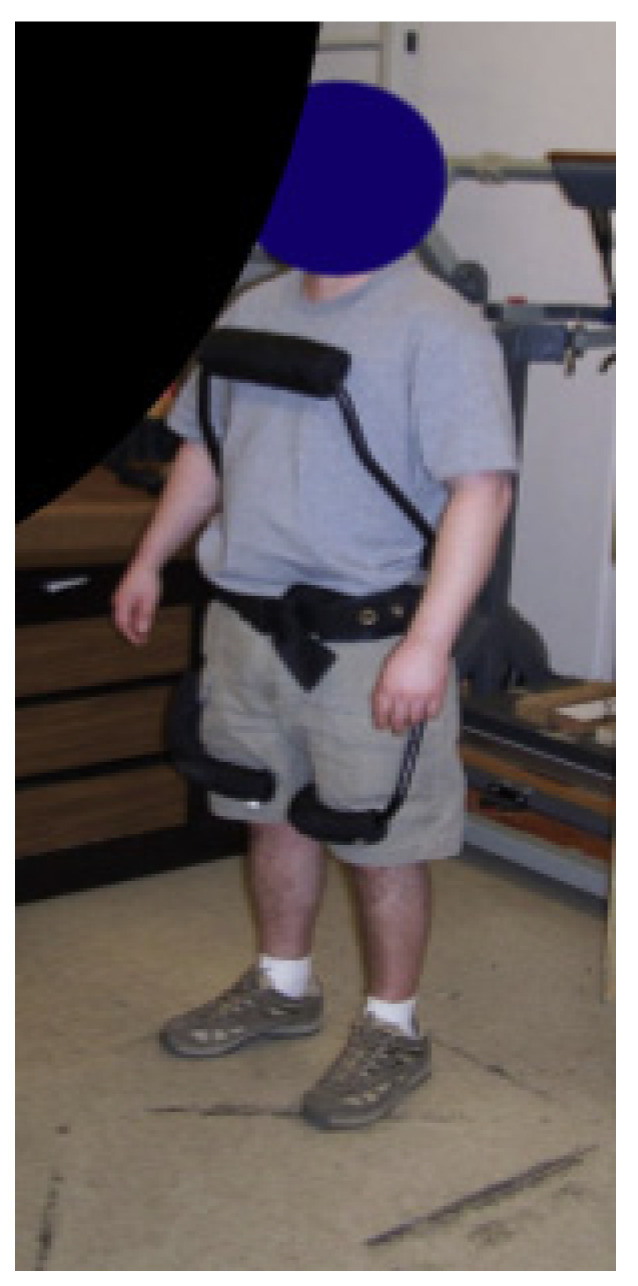
The BNDR.

The BNDR (developed by Limbic Systems in Ventura, CA) is a design that hangs on a waist belt when the user is standing erect and connects to a stiff anterior chest piece and a stiff anterior thigh piece during torso or hip flexions. Torsional springs connect the upper segment with each lower segment. The user’s weight, anthropometry, and stiffness of the device determine the amount of extensor movement provided by the device. This design was found to reduce torso flexion in stooped lifting (lifting with knees in full extension) [58] (see Figure 13). A 13.5% reduction of lumbar spinal compressive force and a 12.5% reduction of anterior-posterior shearing forces at the L5/S1 level were recorded. Together with the 13.7% reduction of posterior lumbar spinal muscle activities during the application of BNDR, the results were mainly attributed to the device’s ability to limit torso flexion [59,60].

**Figure 14:**
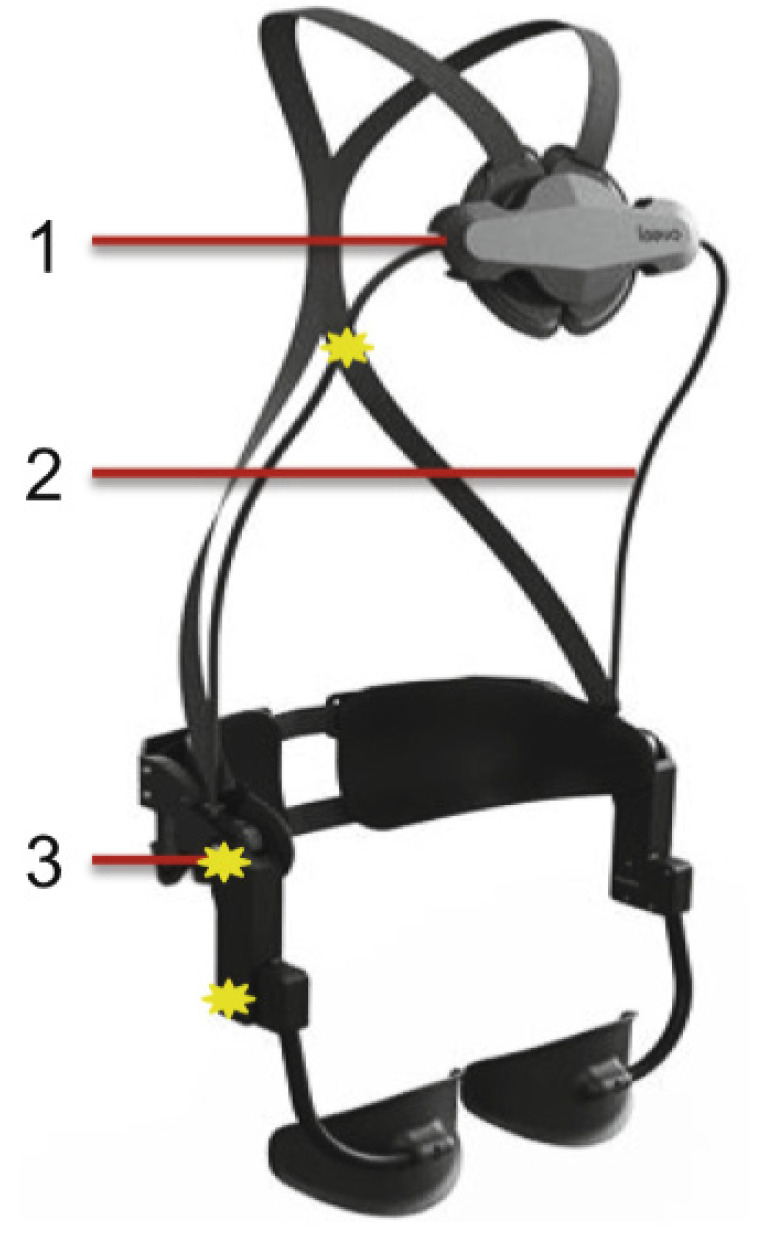
The LAEVO. 1: rotational chest pad 2: flexible beam 3: spring loaded joint.

The LAEVO is a passive exoskeleton that was designed for allied health professionals to reduce incidences of lower back injuries during heavy load carrying and lifting, such as for patient transfer. This design consists of two chest pads, a back pad, and two upper thigh pads. These pads are connected laterally with springs, with the intention to transfer forces from the lower back to the chest and thigh pads. Results have shown that there is a 35%–38% reduction in the posterior spinal muscle EMG activity while using this device for assembly tasks. However, users reported discomfort in the chest region [61] (see Figure 14).

**Figure 15:**
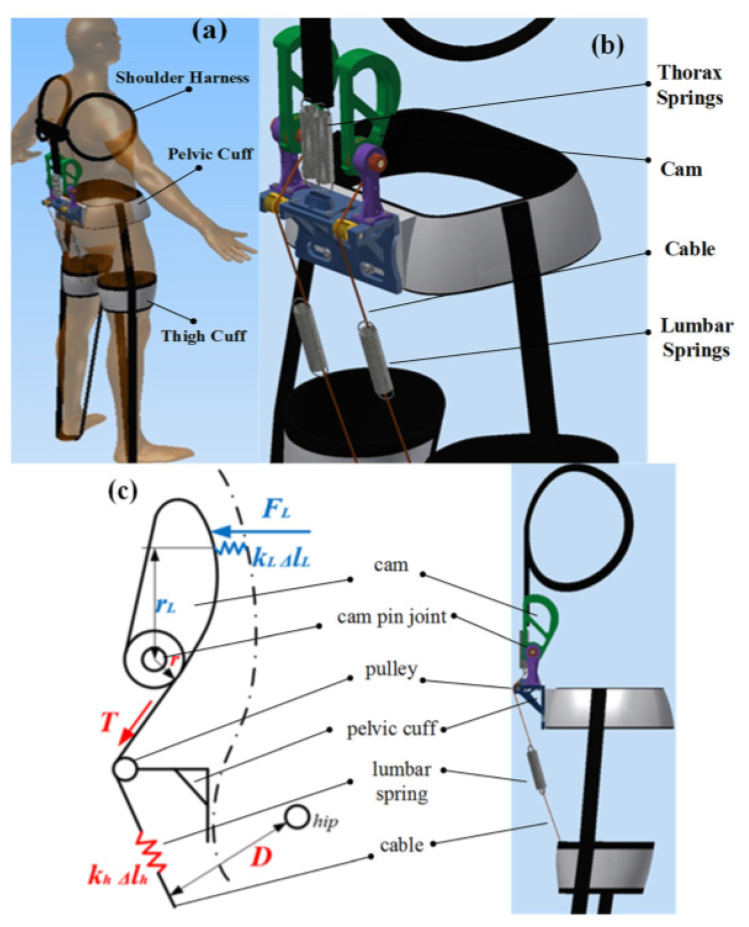
(**a**) dummy user wearing exoskeleton, (**b**) zoomed-in detail view, and (**c**) the cable-tension mechanism of applying the spring pushing force on the human lumbar region.

Another passive spine exoskeleton concept was designed to provide an alternate load pathway to the human spine in an effort to reduce stress on the upper body and therefore mitigate back and shoulder injuries. In order to prevent lower back pain and injury, a group of researchers designed a passive spinal brace, with the aim to reduce loading on the lower back during flexion and extension [50]. The mechanical design of this passive exoskeleton is based on the push–pull strategy of external assistance. A pair of extension springs at the thoracolumbar region is utilized to link the pelvic cuff with the shoulder harness to hold back the upper torso during lumbar spine flexion. The pushing force is generated by employing a cable-tension mechanism connected to another extension spring (lumbar spring) that is attached to the thigh cuff. Recorded posterior spinal muscle EMGs confirmed a reduction by up to 20% at the lumbar region and 50% at the thoracic region in the 14 tested subjects. The 3D-printed fabricated prototype (using mainly acrylonitrile butadiene styrene plastic with steel rods for reinforcement) weighs 3.0 kg including all components, which was reported to be less than 5% of the total body weight for all of the subjects tested in this design [50] (see Figure 15). However, this exoskeleton design does not allow lateral bending or rotational movements of the spine. Ideally, the total component weight should be less than 3% of the total body weight [62].

**Figure 16:**
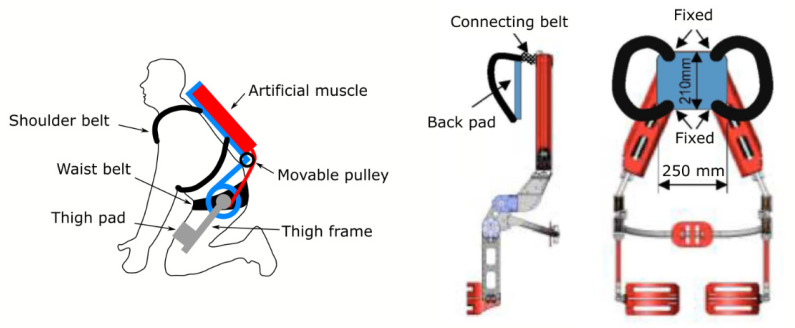
The back support muscle suit (Copyright Springer Nature).

A recent paper [63] pointed out that most of the exoskeletons with the aim of providing lower back support actually do not account for the fact that sagittal plane flexion and extension are dependent on both the movements of the lumbar joints and hips joints. With this in mind, the Back-Support Muscle Suit device has an additional joint adjacent to the hip joints, which is placed at the bottom of the back for lumbar support. The Back-Support Muscle Suit uses McKibben artificial muscles as actuators that are connected to the hip pulley as the center of rotation. This design was developed to support the forward bending of the upper body during load lifting. The contractive force of the actuator is converted to the turning force which extends the upper body. Thigh and back support pads are utilized for the reactive force against the upper body lifting. As reported, this design can reduce posterior spinal muscle EMG up to 40% during weightlifting tasks (see Figure 16).

**Figure 17:**
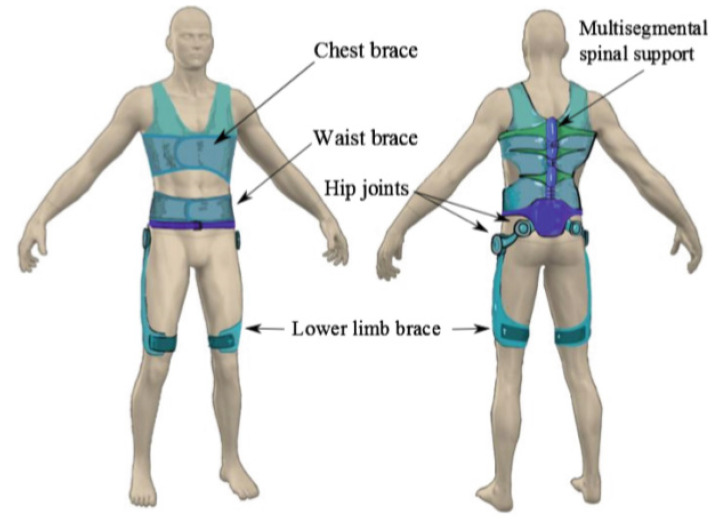
The SPEXOR design (Copyright Springer Nature).

Lower back spinal exoskeletons allow a large range of motion, but the rest of the trunk is not adequately supported. With this in mind, the SPEXOR design, a passive spinal exoskeleton that achieves a similar range of motion to a human lumbar spine of up to 60° in the sagittal plane, was developed to address this need with a passive back support that consists of an elastic mechanism. This mechanism is comprised of flexible beams that run parallel to the spine, providing a large range of motion and lowering the peak torque requirements around the lumbosacral (L5/S1) joint. It also consists of a compensating hip module and a passive hip torque source [51] (see Figure 17).

**Figure 18:**
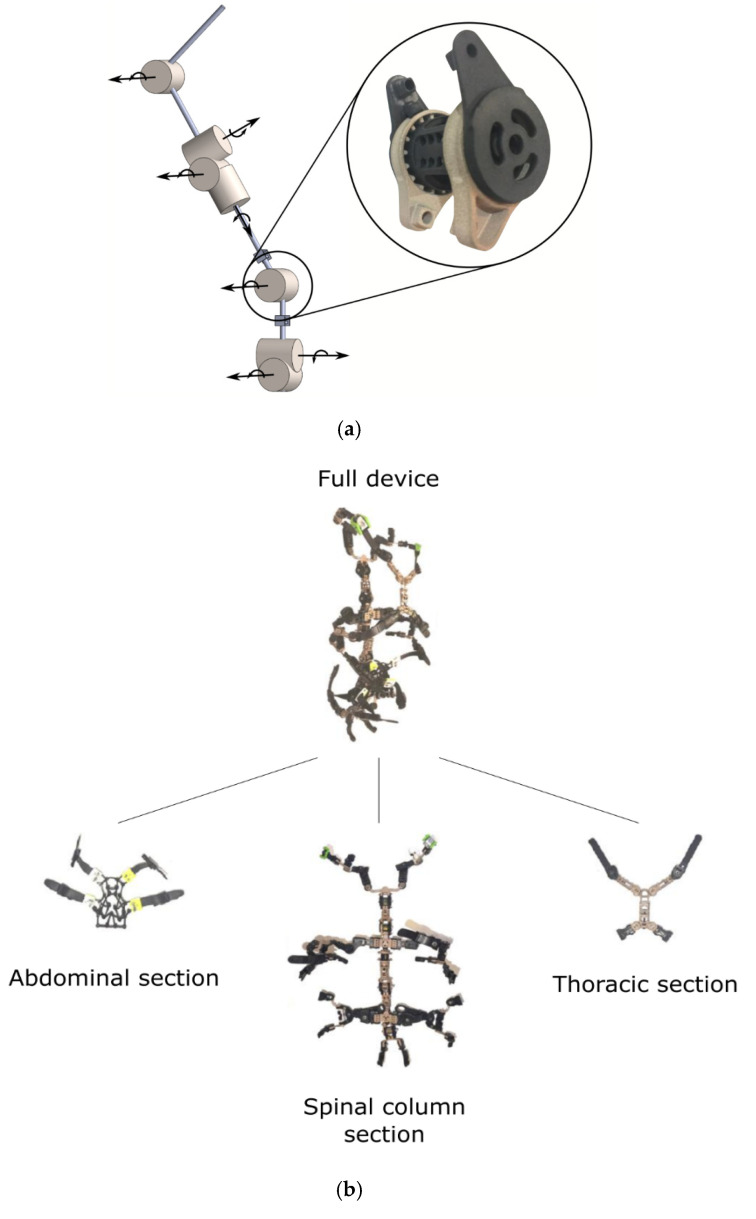
(**a**) The viscoelastic couplings (**b**) the outlook of the werable device.

Most recently, a group of researchers have developed a prototype of articulated spinal exoskeleton where the compression, resistance, and range of motions can be adjusted. The first aim of this device (see Figure 18) was to record and evaluate the interactions between mechanical aspects (compression, resistance, ROM) affecting the human body during different activities, based on the fact that different designs of the traditional rigid spinal braces have a different effect on the body. This particular exoskeleton prototype is a variable-resistance thoracic lumbar sacral (TLS) exoskeleton brace. The main core and column of this exoskeleton are equipped with viscoelastic couplings, which correspond to the lumbar L5, sacral S1, L2-L3, and thoracic T12-L1 and T7-T8 level of the user’s spine. This allows independent control of each joint’s resistance and range of motion. This core column is then connected to the front parts of the exoskeleton using detachable straps to allow different user body habitats. This prototype exoskeleton has already been shown to reduce peak EMG of the posterior spinal muscles by 30%. Preliminary test results have also concluded that adjusting a spinal exoskeleton’s configuration for different tasks by using sensors and actuators would be able to provide more effective support and augmentation for the user [53] (see Figure 18).

**Figure 19:**
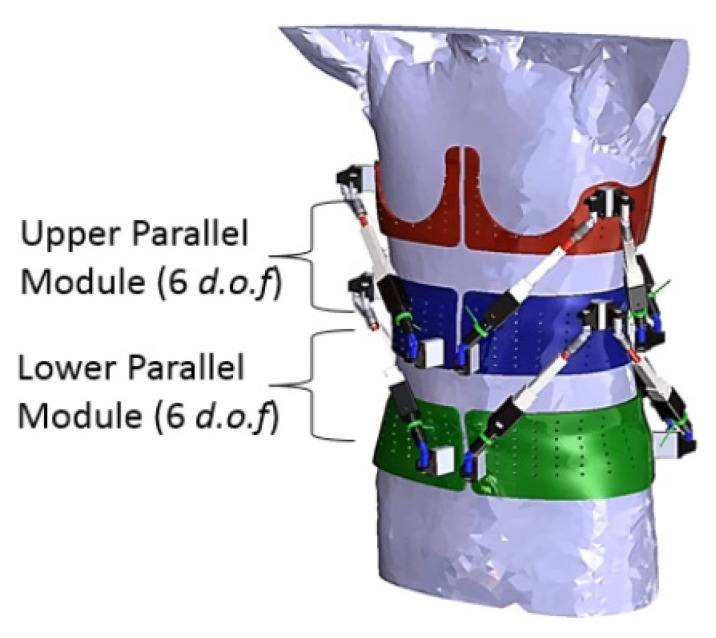
The RoSE.

There are also spinal exoskeletons designed for measurement of spinal parameters. The Robotic Spine Exoskeleton (RoSE) is a dynamic spine brace that looks at in vivo measurements of torso stiffness and characterizes the three-dimensional stiffness of the human torso. Earlier studies used cadavers, which by definition do not provide a dynamic picture. The RoSE measures and modulates the position and forces in all six degrees of freedom in specific regions of the torso. It provides three-dimensional displacements at certain cross sections of the human torso and measures the forces and moments exerted on the torso. Alternatively, the three-dimensional forces on the torso can be controlled while simultaneously measuring and limiting the positions/orientations of the torso [64] (see Figure 19).

(ii) Taxonomy of Devices

The following table, Table 1, summarizes the taxonomy of current devices.

The RoSE spinal exoskeleton was designed specifically for patients with fixed deformities of the spinal column with pronounced asymmetry. It allows measurement of curve-specific forces and moments exerted on the body during long-term bracing treatments, in patients with scoliosis for example. The direct application of a similar device on patients recovering from spinal column injuries or fractures will not be appropriate, as the underlying pathologies are different and the design was also not intended for fracture-related spinal injuries. However, measuring the spine column stiffness could be a useful input to be taken into account when designing a new spinal wearable robot for OSF patients.

Of note, there are currently no spinal soft exo-suit designs with active devices or actuators.

The table below (Table 2) summarizes the current wearable spinal robots.

## 4. Design Considerations for Wearable Spinal Robots

(a) Lumbar spinal forces

When individuals combine forward bending and lateral bending of their torso, the compressive forces exerted on the posterior column of the spine (the facet joints especially) increase. Loss of symmetry during lifting and lowering tasks is more likely to occur, which may result in high loads on an individual’s lumbar spine and create spinal torsion.

A recent study which evaluated the effect of an industrial exoskeleton on muscle activity and perceived musculoskeletal effort concluded that exoskeleton significantly reduced back muscle activity (12–15%) and perceived trunk effort (9.5–11.4%), implying reduced lower back loading. Additionally, the exoskeleton assisted with hip extensor torque as evidence of the significantly decreased biceps femoris muscle activity (5%) [65].

Prolonged static loading could still create a fatigue injury mechanism due to low but prolonged (>2 h) muscle contraction and/or flexed postures of the spine. In a study that looked at lower back joint loading during standing and sitting, it concluded that standing appears to be a good rest from sitting, as a change in lumbar spine posture and shift in loading may rest the posture-dependent passive tissues [66]. Therefore, periodic standing could prevent further strain and injuries to the posterior spinal muscles, which is crucial to pain relief and overall spinal support in OSF patients. This supports the concept of a wearable spinal robot in conservatively managed OSF patients, as it will not only provide spinal axial support, but it also allows earlier mobilization for this group of patients, which may prevent known complications of spinal muscular atrophy. A previous study evaluated the average reaction force against a backrest as 387 ± 28 N [67]. This range may provide a realistic target range for acceptable user comfort of the wearable spinal robot.

One study looked at the effects of carrying a backpack while walking. A disproportionate relationship in trunk muscle activation and lumbar joint loading between light and heavy backpack carriage weight was noted. The study revealed that a load of 3% of body weight will reduce the peak lumbosacral compression force, and a back load of 10% of body weight induced the most critical changes in the load-bearing strategy of the lumbar spine [62,68].

(b) Materials and Wearability

Availability of lightweight materials and new technologies in sensing and actuating enable the development of the next generation of exoskeletons. Conventional exoskeletons consist of a rigid and thick framework of linkages to support the operator’s weight, coupled to the body at select locations via pads, straps, or other interface techniques. Their shape is also difficult to universally fit to various bodies. Titanium is more ideal in terms of strength and weight for metal frames in exoskeletons but is also substantially more expensive. Titanium alloys have a specific strength (the ratio of a material’s yield strength to density) of 44–278 Nm/kg, where the maximum specific strength is 67% higher than that of aluminum alloys with 9–166 Nm/kg [69]. As the wearer flexes or extends their biological joints, these rigid links add considerable inertia to movement, which must be overcome by motors or by the user [70]. Static misalignment of the biological and exoskeleton joints can result in dynamic misalignments of up to 10 cm during normal movement, causing pain and injury to users [71]. Tight straps or skin adhesives are used to maintain the position of wearable devices; however, they cause user discomfort quickly.

Consequently, much research is being conducted on improving wearability, such as the recent work on developing active soft orthotic systems that can be tightly fitted. However, these devices are designed to augment muscle function by applying tension at the wearer’s joints along the direction of muscles, and therefore, they cannot support any device weight.

More recent designs, such as the prototyped articulated TLS spinal exoskeleton mentioned previously, use a combination of 3D-printed metal and nylon components for the couplings and connecting segments. These are surrounded by a flexible housing which is made of a combination of 90A urethane, viscoelastic material (sorbothane), and foam (similar to that used in shoe insoles). As a result, this prototype exoskeleton reportedly only weighs a total of 5 pounds [53].

(c) Recent Design Concepts for Exoskeletons

These recent design concepts are not classified under the current spinal exoskeletons.

**Figure 20:**
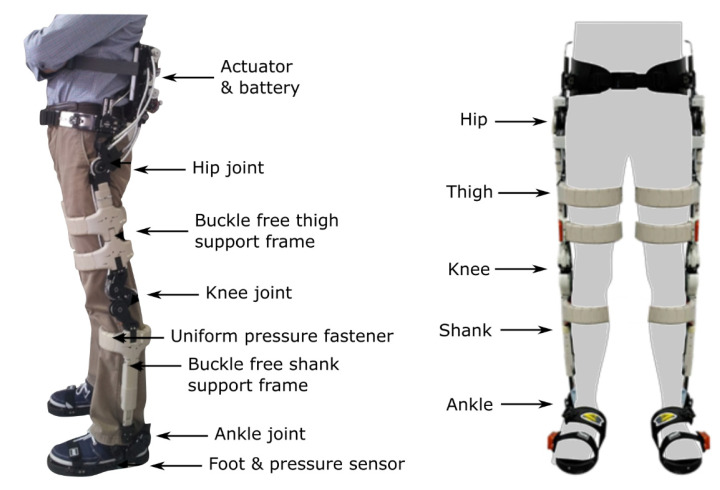
The S-assist device design. Left: lateral view. Right: view from the front.

The S-assist device concept was developed as a buckle-free flexible support frame design which is attached to the hips and lower limbs of the wearer. This frame can bend freely, following the natural curvature of the wearer’s lower body and supporting vertical loads without buckling during bending. This same design pulls the extremity of a human body by wire directly, without load-support frames, to assist the joint torque using tendon-driven actuators, with actuators located in a backpack component [72]. However, despite it being one of the lightest weight designs, the mass of the whole S-Assist prototype still weighs about 14.5 kg (inclusive of actuators and batteries). The batteries can support 60 min of daily activities including walking and sitting-to-standing (see Figure 20).

**Figure 21:**
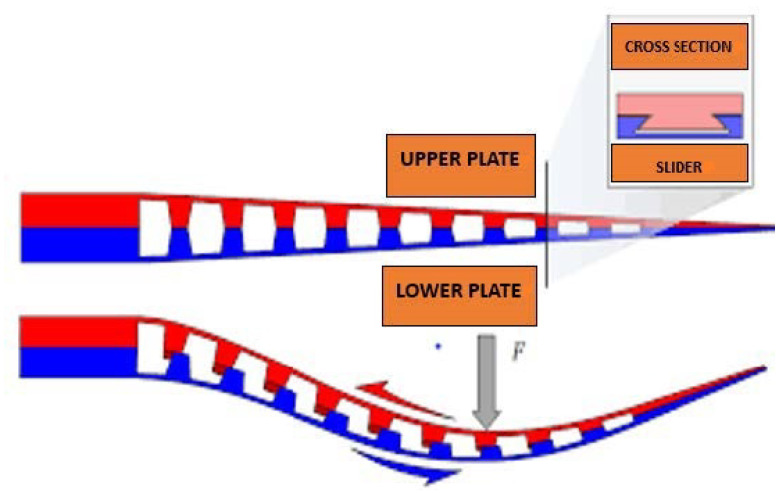
The novel concept of the flexible sliding thigh frame.

A novel concept of a flexible sliding thigh frame for a gait-enhancing mechatronic system has been designed. This is a flexible passive lower limb exoskeleton. The frames of this device are flexible, so that they can be tightly fitted to the wearer’s body and can transmit forces with high stiffness to the designated points, and they are optimized to provide rigidity only in the areas where it is necessary. This flexible exoskeleton is slim and relatively lightweight, with its overall thickness not more than 3cm and weighing only 2.6 kg. Hence, it can be worn readily and comfortably inside clothes. It is capable of transmitting the assistance torque like other exoskeletons without loading the joints and anchor points. The flexible sliding thigh frame uses the same model developed from the mechanical properties of fin rays, a bilaminar structure with the two halves of each ray sliding past each other [73]. This design has essentially made use of all the advantages of exoskeletons with soft orthotics (see Figure 21).

These recent soft exoskeleton designs mentioned are mostly designed for lower limb applications and are mainly directed towards enhancing gait and limb functions of the elderly. To date, there is no such concept applied to a spinal exoskeleton. At the current state of robotic technology, the implementation of a robotic lower-limb exoskeleton capable of biological levels of joint torque and velocity will likely introduce non-negligible mass, rotational inertia, and possibly joint friction. The effectiveness of a soft exo-suit passive robotic spine brace concept is more likely to benefit patients who have suffered from painful OSF, who already may have a certain degree of baseline back pain from spondylosis of the spine (wear and tear due to aging).

(d) Sensors and Controls of Exoskeletons

Exoskeleton designs may incorporate at least some form of mechanical sensor to help regulate position, force, or torque. Effective control strategies are essential for the exoskeleton to work seamlessly with the human body. Common strategies include position control and impedance control. A recent review paper for lower limb exoskeletons had classified assistive controllers into seven groups including: sensitivity amplification, predefined gait trajectory control, model-based control, adaptive oscillator-based control, fuzzy control, predefined action based on gait pattern, and hybrid assistive strategy [74]. Of these, predefined trajectory and model-based control strategies are most commonly adopted [75]. Position-based control is more likely to be used in assisting human locomotion, for which it applies a set of predefined joint angle trajectories. Impedance control could be ideal for a spine brace for patients with OSF; the more the subject deviates from the desired trajectory, the more assistance the rehabilitation device delivers to guide the user back to the desired trajectory, which increases user involvement during training.

Series elastic actuators (SEA) facilitate torque control in some of the lower limb exoskeletons. This type of actuator has a spring-like element in series with the actuator. By measuring the extension and impression of the springs, the SEAs transform the torque/force control problem into a position control problem using their compliance [76]. This facilitates a torque-based control strategy. In addition, these actuators increase user safety and comfort, which is important in applications directly involving human interaction. The spring acts as a buffer for impact and reduces the actuator inertia felt by the user, and springs can also be used to store energy.

EMG signals provide indication for muscle activation and are measured by attaching electrodes to the skin above the desired muscle to be monitored. There are few examples for exoskeletons using EMG signals as feedback and control [77]. The HAL is one of the few commercial devices controlled by neural signals from the muscles (EMG). However, EMG signals may vary between individuals and can be affected by electrode placement and muscle fatigue. Accelerometers and gyroscopes are occasionally included but have not been well documented as being critical nor an improvement to control schemes. The exact control algorithms of many commercial exoskeleton devices are not well documented. Performance comparisons between controllers are rare in the literature, and a single control strategy is typically developed for each individual design.

Neural or brain recordings have not yet been used for commercial exoskeleton controllers to date, but this could be the trend in future development. So far, there have been few studies that specifically account for environmental conditions within their control architecture, but this is expected to change as more of these devices begin to operate in unstructured, real-world environments.

## 5. Discussion

Osteoporotic spine fractures (OSF) are common sequelae of osteoporosis. OSF are directly correlated with increasing age and incidence of osteoporosis. Up to 49 million individuals met the World Health Organization (WHO) osteoporosis criteria in a number of industrialized countries in North America and Europe, as well as in Japan and Australia [78]. They most commonly occur among post-menopausal women. Bone density of the vertebral column decreases steadily with age, with elderly women having lost almost half of their axial bone mass by the time they reach their eighties. One in two women and one in five men over 50 years of age will suffer an osteoporotic fracture. During the year 2000, there were an estimated 9 million new fragility fractures worldwide, of which 1.4 million were symptomatic vertebral fractures.

OSF are treated conservatively or surgically. Associated acute pain, chronic disabilities, and progressive deformities are well documented. Conservative measures include a combination of initial bed rest, analgesia, early physiotherapy, early rehabilitation, and a spinal brace (orthosis).

Spinal bracing is commonly used for symptomatic management of OSF. One randomized trial on the 6-month use of a thoracolumbar orthoses (TLO) brace for osteoporotic compression fractures found improvement in trunk muscle strength, posture, and body height amongst the treatment group and ultimately a better quality of life and ability to perform activities of daily living (ADL). Traditional spinal braces aim to maintain the neutral spinal alignment and limit flexion of the spine, thus reducing axial loading on the fractured vertebra. In addition, the brace allows for less fatigue of the posterior spinal muscles and provides the relief of muscle spasms and pain as a result. Early (within a few days of injury, if possible) mobilization of patients with conservatively treated OSF is recommended, as it will significantly avoid the complications of prolonged bed rest [79].

Downfalls of traditional rigid spinal braces are well known, and they include patient discomfort, which will lead to a decrease in compliance. These patients, typically elderly and frail, are at risk for skin breakdown if the brace edges are not carefully padded. In addition, a brace that is too restrictive may impede the patient’s respiratory volume, leading to chest infections. Finally, with prolonged periods of bracing, there is potential for deconditioning and atrophy of the trunk and paraspinal muscles.

With our current knowledge of spinal braces for conservatively managed OSF, a semi-rigid backpack or bell-style TLO could reduce pain and significantly improve early participation in ADLs.

Exoskeletons are developed to passively assist and actively augment human movements with different types of actuators. Flexible, versatile spinal exoskeletons were designed to better support the spine. More recent devices have started to emphasize the function of a compliant spine in movements and implement flexible structures in their designs. As new technologies enable the development of motorized wearable exoskeletons, several types have been introduced into the medical field application, such as in post-spinal cord injury rehabilitation groups, for example. Although several passive devices have been developed to prevent lower back pain for manual labor work, many of their aspects and functionalities, as well as the practicalities, have yet to be improved.

Despite the rising interest in the spinal exoskeletons and their related publications and research, these exoskeletons are still not widely adopted for routine daily uses, and it seems that a large-scale implementation of spinal exoskeletons in the medical and industrial fields will not happen in the near future. One main reason is the level of user discomfort from mounting or wearing the exoskeleton, as even minimal operator discomfort may degrade user experience and add to reluctance to continue to adapt to the latest technology. The other reason which limits the popularization of exoskeletons is the stigma of social rejection associated with wearing an exoskeleton. The psychological barrier for the operator wearing an exoskeleton arises from the idea of being identified as disabled, as most people prefer to conceal their physical weakness when in public.

A review paper recently concluded that there is still a need to further develop lightweight exoskeletons compatible with operators. Some key technical issues that must be addressed include the design of actuators and artificial muscles, fast and effective control loops, the anthropometric fit, and battery life [45].

As discussed above, there are well-known, documented problems with regards to the daily application of traditional spinal braces for OSF. Rigid spine braces are commonly applied to select groups of patients. In post trauma patients (including those with OSF), spinal braces can prevent overt movements of the spine, which may result in severe pain, and provide spinal support. Bracing is a low risk and cost-effective method to treat post-traumatic thoracolumbar fractures. Post-operative patients can be placed on a spinal brace after spine surgery to reduce movements to allow proper spinal fusion and proper spinal protection in the post-operative periods. Patients with certain degrees of spinal scoliosis and kyphosis are sometimes placed in a spinal brace to help prevent progression of the curves.

To date, there are only a few exoskeleton designs that are targeted at the spine or lower back alone. Of the few which are, these focused mostly on the assistance of the trunk flexion/extension and functional movements. There is no specific wearable robotic spinal brace targeted to assist patients with OSF.

(i) Shortcomings and Limitations of Current Spinal Exoskeletons

As we have discussed above, below is a list of the considered limitations of the current spinal exoskeletons and the suggestions for the design of a wearable spinal robot for OSF (Table 3, see below).

(ii) Suggested design guidelines

A successful spinal exoskeleton for OSF patients will require adequate support at the sagittal, axial, and coronal planes to effectively reduce an individual’s lumbar spinal load and to avoid losing symmetry during ROM. Wearable spinal robots are designed for OSF patients with an acute vertebral fracture (spinal injury), which is a contrast to the other spinal exoskeletons developed to mainly support and augment the lumbar spine to prevent lower back pain. The main aim of a wearable robotic brace is to support OSF patients for engagement of early activities and rehabilitation. At the current state of robotic technology, the implementation of a robotic spine brace capable of actively actuating the biological levels of joint torque and velocity will likely introduce non-negligible mass, rotational inertia, and possibly joint friction. The design of this type of spinal exoskeleton should be a passive mechanism capable of supporting the forces generated for flexion/extension as well as rotation. This translates to most of the usual activities for those suffering from OSF, including sitting up, getting out of bed, transferring from chair to chair, and slow to moderate pace walking. As discussed above, although no extra muscle contractions are required during full flexion-relaxation, the robotic spinal brace should bear forces of approximately 700 N at full range flexion-relaxation to maintain equilibrium. This force is usually generated by the passive tissues, tendons, and ligaments in healthy non-injured individuals. In contrast to the bulky actuators used in other designs, springs, elastic bands, and tension cables should be used in this wearable robotic spine brace. A previous study evaluated the average reaction force against a backrest as 387 ± 28 N. This range may provide a realistic target range for acceptable user comfort of the wearable robotic spine brace to be designed. Thus, the spinal wearable robotic brace for OSF should adapt a soft exo-suit concept with passive lightweight devices for lumbar spinal support.

A novel plug-and-play concept can be used for attachment of external actuators and power supply. In a recent study, IMU sensors were used as sensing tools of the human torso motion range, with algorithms developed to analyze the acquired data, characterizing human torso behavior and motion performances. The early experiments from this study showed that the human torso plays an important part in carrying payloads and in balancing the human body during natural operations [80].

Attachment of IMUs with impedance control could be ideal for a spine brace for OSF patients; the more the subject deviates from the desired trajectory, the more assistance the rehabilitation device delivers to guide the user back to the desired trajectory, which increases user involvement during training.

The important functions addressed by wearable spinal robotics when applied to OSF patients is summarized by the table below. Based on our review of the literature, the specifications were set for the design of a conceptual wearable spinal robot for patients with OSF.

The main aim for a wearable robotic spinal brace in this group of patients will consist of two main domains: reduce spinal bone stress and improve back muscle support. This will lead to back pain reduction, improved posture, and improved overall mobility. As mentioned before, early mobilization is important for management of patients with OSF as it reduces the chance of developing complications related to their immobile state.

The table below summarizes the essential considerations in an effective medical-grade wearable robotic brace that will benefit patients with OSF (Table 4, see below).

## 6. Conclusions

After a thorough review of current robotic technologies, a lightweight spinal wearable robot (as a type of spinal exoskeleton) specifically designed for patients with OSF should be helpful with their recovery, provide spinal support, and reduce back muscle efforts and intervertebral reaction torques during a range of sagittal plane spine flexion/extensions. This would imply augmenting the function of the posterior spinal muscles to maintain global sagittal postural balance. As a result, the spinal wearable robotic brace should prevent acute worsening of the morphology of the fracture, while a certain degree of spinal movements will be allowed. Patients may potentially engage in more activities earlier at rehabilitation, and the potential complications related to the traditional rigid braces will be reduced. The wearable robotic brace may also promote early engagement of active rehabilitation and early return to communities for OSF patients with increased quality of life, while avoiding progressive kyphotic deformity, chronic back pain, and the potential complications related to the traditional rigid braces.

## Figures and Tables

**Table 1:** Summary of the taxonomy of current devices.

Current Devices	Soft Exo-Suits	Rigid Exoskeletons
**Passive Device**	LAEVO (Springs) PLAD (Elastic Bands) SSL (Elastic Bands) Passive Spine Exoskeleton (Springs & Tension Cables)	SPEXOR (Flexible Beams) BNDR (Stiff Torsional Springs) TLS Exoskeleton (Viscoelastic Couplings)
**Active Device**	N/A	Muscle Suit (McKibben Muscle)

Current wearable spinal exoskeletons.

**Table 2:** Summary of the current wearable spinal robots.

Current Wearable Spinal Robots	Original Intention of Design	Active Device	Passive Devices: Soft Exo-Suits	Passive Devices: Rigid Exoskeletons	Unique Difference in the Design	How Does It Help
Muscle Suit	Industrial load carrying, reduce lower back strain	McKibben muscle	-	-	Only wearable spinal exoskeleton with active actuator	Up to 40% reduction in EMGs of posterior spinal muscles at weight-lifting tasks
TLS exoskeleton **	Industrial load carrying, reduce lower back strain	-	-	Viscoelastic couplings	Allows independent control of each joint’s resistance and range of motion at the thoracolumbar spine	Up to 30% reduction in EMGs of posterior spinal muscles
SPEXOR	Industrial and commercial load carrying, reduce lower back strain, lower back pain prevention	-	-	Flexible beams	Aside from flexible lower back support, it also consists of compensating hip module and a passive hip torque source	Awaiting official test results
BNDR	Industrial load carrying, reduce lower back strain	-	-	Stiff torsional springs	Consists of stiff anterior chest and anterior thigh piece to limit torso or hip flexions	13.5% reduction of lumbar spinal compressive force and 12.5% reduction of anterior-posterior shearing forces at the L5/S1 level
LAEVO	Industrial load carrying, reduce lower back strain	-	Springs	-	Transfer forces from the lower back to the chest and thigh pads	35–38% reduction in the posterior spinal muscle EMGs at assembly tasks
PLAD	Industrial load carrying, reduce lower back strain	-	Elastic bands	-	One of the earlier designs—transferring of load from lower back to lower limbs	Up to 40% reduction of posterior spinal muscle EMGs at lifting tasks, and 23–29% reduction of lumbar spine compressive forces
SSL	Medical nursing care (patient transfer), reduce lower back strain	-	Elastic bands	-	Additional elastic belt around the torso for stabilization during flexion and extension of lower back	24.4% reduction in EMGs of the posterior lumbar spinal muscles
Passive Spine Exoskeleton *	For prevention to reduce lower back and shoulder injuries during load carrying	-	Springs and tension cables	-	Based on the push–pull strategy of external assistance	20% reduction in EMGs of posterior spinal muscles
RoSE	Research measures 3D stiffness of torso	-	-	-	-	-

TLS: Thoracolumbar sacral. SPEXOR: BNDR: Bending Non-Demand Return. LAEVO: note, comes from Latin word ‘Levo’, which means enlighten, floating and reducing. PLAD: Personal Lift Augmentation Device. SSL: Smart Suit Lite. RoSE: Robotic Spine Exoskeleton. EMG—Electromyography * Design Concept only ** Prototype.

**Table 3:** Limitations and possible improvement strategy for designing a wearable spinal robot for OSF.

Limitations	Possible Improvement Strategy
Psychological fear of social isolation and being labelled as “disabled”	Wearable exo-suit designs, with soft tight fitting, stretchable materials like elastomers. External batteries (power sources)/ actuators can employ a plug-and-play design when required.
Not accounting for the contribution of hip joints during flexion and extension of spine	Provide adequate thigh and lumbar lower back support reactive forces. Actuators acting on hip as the center of rotation for active assistance of lumbar flexion extension a compensating hip module.
Overall weight of the wearable exoskeleton	Ideally < 3% of total body weight, in order to reduce peak lumbosacral compression forces. Carbon fiber could be the ideal material. Radiolucent material ideal for X-ray films.
Unpredictable forces generated from actuators	Not desirable in injured bony spinal vertebrae for OSF.
Lack of detection of spinal parameters	Attachment of lightweight IMUs to the robotic spinal brace for impedance control for OSF patients.

**Table 4:** Essential considerations in an effective medical-grade wearable robotic brace for OSF patients.

Kinematics Rom Per DoF	Lumbar Spine ROM [6]: Flexion 40–60 Degrees Extension 20–35 Degrees Lateral Bending 15–20 Degrees Axial Rotation 3–18 Degrees
**Ergonomics** Max weight of device Body attachment Materials	< 3% body weight [62] Upper body to hips Soft materials as paddings for comfortable fit Lightweight stiff materials for the main framework for effective force transmission
**Actuation and Control** Active DoF Passive DoF Forces to be generated Actuation and Power Supply Sensors Controls	Flexion and extension Prevention and limitation of lateral bending, rotation (most pain generation from rotation after spinal fracture) At least 300N (the mean extensor strength of the back muscles) [8] Ability to resist up to 700N of force at a fully flexed posture [13] Lightweight and with high power-to-weight ratio Portable Lightweight and efficient power supply (e.g., batteries) Orientation sensors to measure inclination of the spine; possibly pressure sensors at the interface between the device and the body, for contact pressure measurement, hence, this can also derive the underlying 3D stiffness of the spinal column. Increase assistance force based on inclination of the spine

## Data Availability

No new data were created or analyzed in this study. Data sharing is not applicable to this article.

## Abbreviations

OSF	Osteoporotic spine fractures
ADLs	Activities of daily living
QOL	Quality of life
ROM	Range of movements
TLSO	Thoracolumbar spinal orthosis
TLS	Thoracolumbar sacral
DOF	Degrees of freedom
BLEEX	Berkeley Lower Extremity Exoskeleton
EMG	Electromyography
HAL	Hybrid Assistive Limb
FDA	Food and Drug Aministration
PLAD	Personal Lift Augmentation Device
Bndr	Bending Non-Demand Return
SSL	Smart Suit Lite
SEA	Series Elastic Actuators
WHO	World Health Organization
